# Negotiating Physical Health: Professional Logics in Community Mental Health Practice

**DOI:** 10.3390/ijerph23040479

**Published:** 2026-04-10

**Authors:** Gesa Pult, Fabian Frank

**Affiliations:** 1 Department of Educational Sciences, University of Education Freiburg, Kunzenweg 21, D-79117 Freiburg, Germany; 2 Department of Social Work, Protestant University of Applied Sciences Freiburg, Bugginger Strade 38, D-79114 Freiburg, Germany; fabian.frank@eh-freiburg.de

**Keywords:** health equity, community mental health, social work, serious mental illness, physical health

## Abstract

**Highlights:**

**Public health relevance—How does this work relate to a public health issue?**

- People with serious mental illness (SMI) experience substantial and largely preventable physical health inequities.
- Community mental health services (CMH) are key everyday settings where these inequities are encountered and potentially addressed.

**Public health significance—Why is this work of significance to public health?**

- The study shows how physical health is interpreted and negotiated within routine CMH practice.
- Five professional logics explain when and how physical health becomes part of everyday support.

**Public health implications—What are the key implications or messages for practitioners, policy makers and/or researchers in public health?**

- Strengthening relational, organisational, and environmental conditions may enable CMH services to address physical health more systematically.
- Clarifying mandates and support structures could help integrate physical-health-related support into psychosocial CMH care.

**Abstract:**

Individuals with serious mental illness (SMI) face profound and largely preventable physical health inequities shaped by social and structural conditions, representing a major public health concern related to avoidable health inequalities. Because many receive everyday support in community mental health (CMH) systems, these services represent a crucial arena for understanding how such inequities are encountered and made sense of in practice. The study examines how physical health is understood within German CMH practice. Five group discussions with 30 CMH workers were analysed using an interpretive qualitative approach. The analysis identified five professional logics through which physical health becomes part of CMH support: trusting relationships that both enable and limit action; psychological stability as a core mandate; physical health positioned between recognition and delegation; fragile motivation combined with an ethics of restraint; and health promotion situated between aspiration and structural constraint. The findings show that helping relationships, everyday environments, and organisational structures create specific conditions for health-related support. Strengthening these interconnected levels may enable CMH to integrate physical health more systematically, offering insights relevant to international CMH contexts facing similar relational and structural challenges.

## 1. Introduction

Health equity is commonly defined as the reduction in systematic and avoidable differences in health between social groups [1]. From this perspective, health is not merely a biomedical state, but a socially and structurally shaped phenomenon, influenced by the distribution of resources, opportunities, and living conditions [2,3,4]. Within public health research, these social determinants of health are widely understood as central drivers of population health and health inequalities [3,5,6]. This framework is particularly relevant for understanding the situation of individuals with serious mental illness (SMI), whose everyday lives are often shaped by structural disadvantage [3], stigma [7], and limited access to responsive health-care systems [8].

Individuals with SMI experience substantially higher rates of chronic physical conditions [9] and a markedly reduced life expectancy of up to 25 years [10,11] compared to the general population. These disparities are largely preventable [9,12] and arise from a combination of medication-related metabolic risks, fragmented health-care provision, and elevated exposure to modifiable risk factors such as smoking and physical inactivity [12].

From a capability perspective, health depends not only on access to services, but on individuals’ real opportunities to transform available resources into meaningful action [13,14]. Applied to individuals with SMI, this perspective highlights that persistent health disparities are less the result of individual deficits than of constrained capabilities shaped by everyday contexts and support structures.

In this context, the concept of health literacy provides an additional perspective on how individuals engage with health. Health literacy refers to people’s knowledge, motivation, and competencies to access, understand, appraise, and apply health-related information in everyday life [15]. For individuals with SMI, health literacy is often shaped by cognitive, social, and structural constraints, making supportive environments and relational forms of engagement particularly important. Within CMH settings, this suggests that supporting physical health is less about delivering standardised interventions and more about enabling individuals to develop and enact health-related capacities within their everyday contexts [16].

A growing body of research shows that improvements in physical health among individuals with SMI are achievable through targeted interventions [17], including physical activity, nutrition, and smoking cessation [17]. Evidence further suggests that intervention impact increases when embedded in everyday-life contexts, adapted to motivational and cognitive needs, and grounded in stable, trusting relationships [18]. Despite this evidence, the implementation of physically oriented health promotion within community mental health (CMH) services remains limited and insufficiently anchored in routine practice [12,19].

Social work, as a psychosocial profession committed to supporting individuals in their interactions with social structures and environments, offers a practice framework that links personal circumstances with broader societal conditions [20]. This orientation aligns closely with relational, everyday-life-based, and context-sensitive approaches that have been identified as particularly promising for supporting physical health among individuals with SMI [18]. With its emphasis on participation, durable helping relationships, and attention to clients’ social environments, social work is well positioned to address health-related needs that are embedded in everyday life [21,22].

Despite these conceptual alignments, social work remains only marginally integrated within many CMH systems and is often confined to organisational or administrative roles rather than recognised as a profession with specific competencies in health-related practice [23]. At the same time, CMH systems vary considerably across countries. In Germany, support for individuals with SMI is primarily organised within a psychosocial and social-participation framework and largely delivered by social workers [24]. These services, typically provided through assisted living and psychosocial support, aim to promote participation, everyday functioning, and quality of life [24], thereby constituting a key setting in which physical health disparities become visible and potentially addressable.

Examining how CMH workers interpret, prioritise, and take up physical health in their daily work can therefore provide important insights into the conditions under which health-related support can be integrated into psychosocial support. Despite its relevance for both the German context and broader international debates on community mental health, the role of social work in addressing physical health has received little systematic empirical attention.

Therefore, the present study addresses the following research questions:

(1) How do CMH workers understand their role in relation to physical health among individuals with SMI?
(2) Which orientation patterns or implicit professional logics shape how CMH workers take up physical-health-related responsibilities in everyday practice?
(3) What do these implicit logics imply for the future development of physical health promotion in CMH setting?

## 2. Method

The following sections describe the qualitative–interpretive design, the recruitment and composition of the group discussion, the procedures of data collection, and the interpretive analytical steps guiding the development of the findings.

### 2.1. Study Design

The study adopted a qualitative design. It is grounded in interpretive traditions that view social reality as constructed through interaction and situated meaning-making, drawing on perspectives from symbolic interactionism [25,26]. Within this design, data were collected at a single point in time in CMH services.

Group discussions were selected because they are particularly suited to making these often implicit constructions and collective meaning structures visible [27,28]. The term group discussion is used here to denote naturally occurring work groups as “real groups” [28] engaging in interaction-based meaning-making, in contrast to more structured focus group formats [28]. This approach differs from focus groups, which typically follow a more structured protocol. In contrast, the present study aims to capture collective meaning-making within existing teams in their everyday professional context.

### 2.2. Participants

The sample comprised German CMH workers who supported individuals with SMI in one large city and two adjacent rural districts. Local CMH providers were invited to take part in group discussions, and interested staff received written information outlining the study’s aims, procedures, and participant rights. Prior to participation, all individuals were additionally provided with verbal information and given the opportunity to ask questions and seek clarification. Informed consent was obtained only after this full briefing, including explicit assurance of the right to withdraw at any time without disadvantage.

Five group discussions were conducted. Each discussion took place with an existing team as a “real group” [28]. The participating organisations differed in provider structure and service type, offering some contextual variation across groups. Ethical approval was granted by the ethics committee of the University of Freiburg (reference: 24-1354-S2), and the study was preregistered on the Open Science Framework [29].

The sample consisted of 30 German CMH workers (26 women, 4 men) aged between 25 and 66 years (M = 46.9; MD = 53.5), all of whom worked in different forms of supported housing, including community-based and residential services as well as family-based supported housing for adults. In all cases, the group discussions were conducted with “real groups”, that is, existing staff teams who work together in their everyday practice [28]. Group sizes varied between 2 and 13 participants (Group 1: *n* = 4; Group 2: *n* = 5; Group 3: *n* = 2; Group 4: *n* = 6; Group 5: *n* = 13).

Participants’ professional backgrounds included social work (*n* = 13) and other pedagogical professions (*n* = 9), as well as special education (*n* = 3), occupational therapy (*n* = 3), psychology (*n* = 1), psychiatric nursing (*n* = 1), and additional non-medical qualifications (*n* = 3). Work experience in their current position ranged from 1 to 35 years (M = 12.3; MD = 8.5).

The names of participating organisations are not disclosed to ensure the anonymity of both organisations and participants, as the use of naturally occurring teams could allow indirect identification.

### 2.3. Data Collection

The five group discussions were conducted between December 2024 and February 2025 in the premises of the participating CMH organisations. Each discussion lasted approximately 50–60 min, was audio-recorded, and moderated by the first author. A semi-structured format was used to balance narrative openness with comparability across groups. The topic guide provided thematic orientation but did not constitute a standardized protocol, allowing participants to shape the discussion through their interaction. The topic guide consisted of open, narrative-oriented questions and short thematic prompts designed to elicit concrete experiences, everyday practices and reflections on physical health in CMH work. It was informed by a preceding systematic review [30] drawing on the COM-B model of behaviour change [31] and served as a flexible sensitising framework.

In line with group discussion approaches [28,32], participants were encouraged to speak freely, to respond directly to one another’s accounts, and to elaborate both convergent and divergent perspectives that emerged in the group. The moderator facilitated interaction and ensured that all participants could contribute without introducing interpretations during the discussion.

The prompts were designed to address the study’s research questions. Key prompts included:

- “What role does health play in the support you provide through CMH services?”;
- “How do you perceive the overall health of your clients?”;
- “How would you describe your clients’ motivation for health-related behavioural change?”;
- “How do you think about targeted physical health promotion for people with SMI, both within and beyond CMH settings?”.

### 2.4. Analysis

All group discussions were transcribed verbatim; identifying information was removed or altered to ensure anonymity. Data analysis followed the Integrative Basic Method [26], developed by German social scientist Jan Kruse. It promotes a comprehensive description of linguistic–communicative phenomena as the basis for intersubjectively comprehensible interpretation. Language is understood as a medium of social interaction and as a tool for self-understanding [26,33].

Analyses followed Kruse’s suggested interpretation steps:

- Step 1: Descriptive fine segmentation: Identified shifts in the flow of the answers by marking structural units anchored in short text fragments, without assigning any interpretive meaning.
- Step 2: Descriptive fine analysis: Interpretation remained on descriptive level. The goal was to produce a precise sequential map of how the utterance unfolds word by word.
- Step 3: Reconstructive fine analysis: The analysis shifted from description to reconstruction, identifying central logics through additional analytical lenses such as agency, metaphor, and positioning grounded in the descriptive analysis.
- Step 4: Comparison segments within one case: After the reconstructive analysis of one segment, we traced for these logics throughout the whole case. Further passages were selected and explored to see whether or not the logic was reaffirmed or challenged, and whether additional logics emerged.
- Step 5: Comparison across cases: Reconstructed logics were compared between group discussions to identify recurring patterns of meaning-making.

Interpretive group work ensured plausibility and transparency and linked openness with methodological control.

To illustrate the analytical procedure, one example may clarify how professional logics were reconstructed. In one segment, participants emphasised the importance of “not pushing too hard” in relation to clients’ engagement with health-related behaviour. In the descriptive steps, this was first identified as a recurring pattern of caution in interaction. In the reconstructive analysis, this pattern was interpreted as reflecting a tension between encouraging change and preserving relational stability. Across cases, similar patterns were identified and compared, contributing to the reconstruction of the professional logic described as an “ethics of restraint”.

## 3. Results

The analysis identified five professional logics across all group discussion, independent of organisational arrangement, that shape the everyday practice of CMH workers. Each professional logic gives rise to specific tensions between meaning horizons that limit the perceived scope of action, particularly in situations marked by contradictory expectations. All quotations from participants were translated from German into English and lightly smoothed for readability.

To situate the findings, a description of the sample is provided below, followed by the five professional logics identified in the analysis: (1) trusting relationships situated between enablement and limits of action; (2) psychological stability as a core professional mandate; (3) physical health between recognition and delegation; (4) fragile client motivation and an ethics of restraint; and (5) health promotion between professional aspiration and structural constraints.

### 3.1. Trusting Relationships Between Enablement and Limits of Action

Across all groups, trusting relationships emerged as the primary medium of support. In participants accounts, relationship is not merely a context for intervention but the condition under which stability, safety, and change become possible:

“*This social component […] this relationship we offer to people, the sense of safety we give them—that’s just invaluable. […] It’s about having someone you can turn to, knowing that you can come with your worries and problems, that you can be who you are, and that you won’t be patronised*”.

(GD_1)

At the same time, this relational foundation is marked by a tension between enablement and professional limits. Trust opens space for engagement and participation, yet it also sets boundaries for what can be enacted. Participants emphasised that trust requires time, emotional presence, and reliability, while simultaneously demanding distance and self-protection to avoid overload:

“*We don’t make decisions for them—we talk with them. […] This attitude of ‘I’m all-knowing, I know what you need and how you feel, and therefore we’ll do it my way’—that’s not what we do*”.

(GD_1)

Clients were described as relying on authentic, consistent contact but as highly sensitive to pressure or paternalism. Professional action therefore involves holding a careful balance: close enough to create safety, but sufficiently distanced to respect autonomy.

This relational logic captures how participants navigate a dual horizon in their everyday practice, one in which possibilities for stability and change are negotiated within the very boundaries that trust both enables and constrains.

### 3.2. Psychological Stability as a Core Professional Mandate—Health Framed Through Protection and Crisis Prevention

Across all groups, psychological stability emerged as the central mandate guiding participants’ everyday practice. Stability is portrayed as the prerequisite for any form of support and as the condition under which participation, wellbeing, and daily functioning become possible. Rather than a fixed state, participants describe it as an ongoing balancing process that must be continually re-established in both everyday routines and potential crises:

“*And I think it’s really important that it’s my job, as a professional service, to look closely, to be there, to advocate for my clients*”.

(GD_2)

Participants see themselves as structuring often unstable daily lives, preventing crises, and securing emotional safety. Within this logic, physical health becomes relevant primarily when it contributes to stabilization—or at least does not jeopardise it. Topics such as exercise, nutrition, or medical care are taken up only when they fit within existing relationships and stabilisation-oriented work processes:

“*It’s about creating some stability within this illness that they have to live their everyday lives with […] about working out, or getting to, some kind of straight line together with them*”.

(GD_5)

A clear tension shapes this logic. Clients are described as vulnerable, exhausted, and easily overwhelmed, yet also as determined in upholding their boundaries. Professional action therefore requires navigating the fine line between safeguarding and respecting autonomy:

“*Some people are paralysed by depression […] and if they want support, you can give it to them. But you have to balance it very carefully: where is the line between ‘I’m offering you help’ and ‘I’m telling you this is how it should be done’*”.

(GD_1)

Taken together, this stabilisation logic forms a central reference point for everyday practice and sets boundaries around when and how physical-health-related topics can be addressed—namely, only when they align with the overarching aim of maintaining psychological stability.

### 3.3. Physical Health Between Recognition and Delegation—Boundary-Drawing as a Professional Positioning

The third professional logic concerns how physical health is positioned within everyday practice. Across all groups, participants described clients’ physical health as limited or impaired, frequently noting overweight, inactivity, chronic pain, and high levels of tobacco use. Such observations were often linked to concern, as physical complaints were seen as reducing quality of life and intersecting with mental illness, poverty, and medication effects:

“*The physical health of many people is really quite poor […] it all comes together: medication, little physical activity, no motivation, and then the pain on top of that*”.

(GD_3)

Despite this awareness, physical health was seldom treated as a distinct field of action. Responsibility was largely attributed to others—general practitioners, psychiatrists, and other therapists—or left to clients themselves. In this logic, physical health is acknowledged as relevant yet located outside the professional remit.

“*If people can’t find a general practitioner or keep getting sent away, I can tell them a hundred times to ‘go see a doctor’—it doesn’t help. The structure just isn’t there*”.

(GD_2)

Delegation thus fulfils a double function: it lowers expectations for which CMH workers feel neither trained nor mandated, and it delineates the boundaries of their professional role. Boundary-drawing appears as a routine aspect of positioning in everyday work—whether to accompany, to step back, or to leave responsibility with clients:

“*I can’t force anyone to go to the doctor […] I can accompany them, but the decision is theirs. And if they say ‘no’, then that’s just how it is*”.

(GD_2)

A tension becomes visible between contrasting meaning horizons. Clients are portrayed as disadvantaged in their physical health and constrained by structural barriers, yet at the same time as exercising agency when declining or postponing health-related action. Because the core mandate is understood as fostering participation and everyday stability rather than providing health-care, physical health occupies an ambivalent position: recognised as important, but often perceived as lying outside legitimate professional scope.

Overall, this boundary-drawing logic positions as relevant but primarily delegated to other systems or to clients’ own responsibility.

### 3.4. Fragile Motivation and an Ethics of Restraint—Negotiating Care, Autonomy, and Self-Protection

Across all groups, a fourth professional logic concerns the fragile and fluctuating nature of clients’ motivation to engage with physical health. Motivation is described as present in principle, yet easily disrupted by mood, symptoms, or everyday burdens:

“*At first, many say, ‘Yeah, it would be good to do something for myself’—but after two weeks, it’s gone again. Then something else comes up, or their mood just drops*”.

(GD_4)

In the discussions, an ethos of caution and restraint emerges as a typical response. Pressure or overly ambitious goals are seen as counterproductive, risking withdrawal rather than engagement. Instead, patience, trust, and low-threshold, situational offers form the basis of supportive action. Within this logic, physical health promotion is understood more as an invitation than a demand:

“*I can give encouragement, but I can’t force anyone […] If I push too hard, I lose them*”.

(GD_1)

Restraint carries a double meaning in the data: it expresses respect for clients’ autonomy and pace, while simultaneously functioning as a protective mechanism for participants themselves. The material shows an ongoing tension between wanting to encourage change and needing to remain within one’s own emotional and professional limits. Engagement is adjusted to clients’ psychological state and capacity for change:

“*I’m not sure how I could support her better so that she takes that first step*”.

(GD_1)

Overall, this logic of restraint appears in the data as both a deliberate stance and a response to uncertainty: a professional form of caution that protects relationship and stability, avoids overload, and acknowledges the limits of one’s influence in health-related change.

### 3.5. Health Promotion Between Professional Aspiration and Structural Constraint—An Institutional Vacuum as a Collective Experience

The fifth professional logic concerns how physical health is situated within the broader organisational context. Across all groups, a strong sense of relevance emerged, alongside a desire to support clients more actively in this area:

“*[…] that we actually know that people with mental illness have a shorter life expectancy, and I feel like it’s somehow a taboo. Like, from us, from the people themselves*”.

(GD_4)

Yet efforts to address physical health were described as largely dependent on individual commitment, rarely secured by organisational structures. Time, space, and budget constraints shaped what was feasible in everyday practice. While initiatives existed, many had lapsed over time or were tied to single individuals rather than embedded institutionally. Cooperation with external organisations was mentioned in some settings, though often with limited effect:

“*If you want to do that, you somehow have to fight for it—time, space, budget, everything*”.

(GD_3)

This produces a clear tension between professional aspiration and structural constraint. Physical health is viewed as socially necessary and central to clients’ wellbeing, yet the organisational environment was repeatedly described as lacking resources, clear responsibilities, and sustained concepts—resulting in what participants framed as an institutional vacuum.

Alongside these structural conditions, professional stance also shapes how health-related practice is taken up. Participants emphasised that support cannot be externally prescribed but must align with clients’ everyday realities and subjective meanings. Such views reflect underlying professional values that differ from conventional health-promotion models; standardised or programme-based interventions were seen as insufficiently aligned with clients’ everyday contexts, needs, and capacities to clients’ needs or capacities. Instead, relational, autonomy-oriented, and everyday-relevant approaches were emphasised:

“*We’d need a way of thinking about health that makes it a joy to care for myself—to feel good about doing what’s good for me, to see caring for my health as something positive, especially for our clients*”.

(GD_4)

Health-promoting activities were described as most successful when embedded in ongoing relationships and everyday support processes. The focus lay less on objective health parameters and more on clients’ lived experience, shaped through small, self-determined steps and gradual change.

Overall, this structural–aspirational logic illustrates how physical health is addressed within organisational limits: it is taken up when it fits into established relational and support routines, yet remains dependent on local conditions and individual initiative in the absence of stable institutional frameworks.

## 4. Discussion

The study set out to explore how German CMH workers understand physical health in the context of their daily work and how they take up physical-health-related responsibilities within their professional mandate. Using group discussions and an interpretive analytical approach, the analysis identified five professional logics through which physical health is negotiated in relationships, everyday routines, and organisational contexts. The Discussion therefore focuses closely on the five empirically derived professional logics, examining how these logics structure everyday decision-making and shape the conditions under which physical health becomes addressable in practice.

In the following, these findings are examined in relation to methodological considerations, core features of social work practice, and the profession’s potential contribution to health equity. The discussion then considers the conditions under which physical-health-related support can be strengthened within community mental health settings.

### 4.1. Methodological Reflections and Limitations

A key strength of this study lies in its qualitative–interpretive design. The use of group discussions with naturally occurring teams provided rich access to shared understandings and implicit interpretive logics that unfold through interaction. The analytic approach, informed by interpretive traditions and operationalised through the Integrative Basic Method [26], allowed for an iterative and fine-grained engagement with the linguistic, interactional and meaning-related dimensions of the data. Drawing on principles associated with documentary–interpretive analysis [28,32] supported the identification of implicit professional logics while remaining closely grounded in participants’ own formulations.

The sample size aligns with established guidance for interpretive qualitative research [27], where the quality of interaction and the emergence of shared meaning structures are central. Including CMH workers from one urban and two adjacent rural areas provided a degree of contextual breadth across organisational settings. While participants differed in formal qualifications, the sample reflected the typical composition of German CMH teams—predominantly social workers or allied psychosocial practitioners. These professions share a comparable practice base in this context, suggesting that the heterogeneity of professional titles is unlikely to have biased the findings.

Several limitations should be noted. The study was conducted within the German CMH system, whose psychosocial and participation-oriented mandate differs from medically anchored CMH models in other countries; transferability to other contexts is therefore limited. In more clinically oriented CMH systems, such as those in the UK or the United States, the identified professional logics may manifest differently, particularly with regard to the balance between psychosocial support and medically defined responsibilities. For example, the tension between relational support and delegated responsibility for physical health may be structured through clearer clinical mandates or more direct integration of health-care tasks. However, such considerations remain speculative and highlight the need for comparative research across different CMH systems. Participation was voluntary, which may have encouraged involvement by individuals with greater interest in health-related topics, introducing possible selection bias. The sample was predominantly female, reflecting the gender distribution commonly found in German social and CMH services [34]. While this may shape orientations towards care, responsibility, and boundary-setting, the study was not designed to systematically analyse gendered differences in professional practice. The extent to which the identified professional logics are influenced by gender therefore remains an open question for future research.

### 4.2. Professional Logics: Social Work as Reflexive and Relationship-Oriented Practice at the Intersection of Mental and Physical Health

The group discussions show that German CMH workers understand securing psychological stability as the core of their professional mandate. Their practice is grounded in relationships shaped by trust, respect, and recognition. Relationship appears not as a technique but as the central space in which professional judgement unfolds. This aligns with social work theories that view professional action as reflexive, requiring practitioners to continually interpret situations, adjust their stance, and decide when intervention or restraint is appropriate [22,35]. Reflexivity thus emerges as a central professional resource in everyday practice.

The findings also reflect the antinomies that characterise social work: navigating proximity and distance, stabilisation and change, care and self-determination [22,36,37]. These tensions are not problems to be resolved but inherent conditions that shape how judgement becomes possible. Within this context, practice unfolds under uncertainty—a widely recognised feature of social work across theoretical traditions [35,36,38]. In the group discussions, German CMH workers describe weighing how to support clients without undermining autonomy, how to invite change without creating pressure, and how to maintain stability amidst everyday challenges.

Seen through this lens, the findings illustrate how physical health becomes folded into these ongoing negotiations of care, autonomy, and stability. Although clients’ physical health is often perceived as impaired, its promotion is not regarded as a core responsibility. Psychological stability and participation guide everyday action, with responsibility for physical health typically delegated to clients or to medical professionals. This reflects a dynamic within the underlying professional antinomies: impulses toward change are moderated to preserve relational stability and respect self-determination.

At the same time, the data reveal developmental potential. The reflexive stance that characterises CMH practice—marked by careful balancing of support and restraint and sensitivity to clients’ pace—provides a foundation for addressing physical health more systematically. Yet, this potential is not realised as routine practice; it remains contingent on individual commitment, situational judgement, and organisational conditions. Extending physical-health-related support in CMH would therefore require drawing on these relational strengths while providing structural support and competencies that allow physical concerns to be integrated without jeopardising psychological stability or professional boundaries.

### 4.3. Social Work as a Profession in CMH Contributing to Health Equity

Health equity requires addressing the social and structural conditions that shape people’s opportunities to achieve health [1]. If health is understood as socially produced [3,4], then reducing health inequalities cannot be the task of medical professions alone. Social work, with its mandate to engage individuals and structures to enhance wellbeing [20], is inherently positioned to contribute to health equity through its person-in-environment perspective [39,40,41]. This orientation understands wellbeing as emerging from the interplay of individuals, relationships, and environments—closely aligning with the realities of community mental health practice [24,42].

The findings of this study show that physical health is supported in ways that reflect this professional perspective. Three competencies emerge as central pathways through which CMH practice can engage with health promotion:

(1) Relationship orientation: Trust-based relationships create a space in which clients can articulate concerns, stabilise routines, and consider health-related steps at their own pace.
(2) Everyday embeddedness: Health topics are taken up when they fit into clients’ daily lives and existing support processes, rather than through externally prescribed programmes.
(3) Reflexivity: Navigating tensions between encouragement and restraint, autonomy and protection, constantly adjusting their involvement to clients’ psychological and social conditions.

These competencies enhance clients’ opportunities to engage with health-related issues within the constraints of their everyday environments. In this sense, the findings resonate with capability-oriented perspectives, which emphasise people’s real opportunities to convert available resources into meaningful action [13,14]. Motivation, meaning-making, and relational stability form part of these opportunities.

Within these boundaries, social work in CMH brings health-relevant capacities that can help reduce avoidable physical health disparities without redefining CMH as a medical service. Rather, health becomes part of everyday support when understood as embedded in clients’ relational, psychological, and social contexts. In this sense, CMH practice contributes not only by addressing structural conditions, but also by supporting individuals’ capacities to engage with and make sense of their own physical health within the constraints of their everyday lives.

### 4.4. Making Health Equity Work: Conditions for Health-Promoting Practice in CMH Social Work

The study shows that German CMH workers do not treat physical health as an isolated goal but as intertwined with everyday coping, psychological stability, and self-determination. Across the five professional logics, health becomes relevant when it can be embedded in trusted relationships, aligned with clients’ routines, and negotiated through reflexive, situational judgement. These findings indicate that health-promoting practice in CMH depends less on stand-alone interventions than on the relational, social, and organisational conditions that enable small, meaningful steps within clients’ everyday environments. From a public health perspective, the findings align with evidence highlighting relational and contextual conditions as important determinants of health and health inequalities, pointing to the role of everyday support systems in shaping opportunities for health-promoting action [3,5,6].

To understand these conditions more systematically, a network perspective offers a useful frame. The five professional logics demonstrate that health-related practice unfolds within multiple, interdependent layers of clients’ lives: the relationships that sustain everyday stability, the social environments that shape opportunities for action, and the organisational structures that enable or constrain support. Drawing on network theory [43,44] and on distinctions between primary, secondary, and tertiary networks in social work [45,46], these layers can be conceptualised as three interconnected levels—personal, social–environmental, and institutional. The findings suggest that extending CMH practice to include physical health requires specific conditions at each of these levels. These conditions are not introduced as abstract extensions, but are analytically derived from the five professional logics and their underlying tensions as reconstructed in the empirical material.

### 4.5. Primary Network Level: Relationship as a Space for Health-Related Reflection

At the primary network level, the helping relationship forms the central space in which health-related concerns can surface. Relational continuity, attentiveness, and reflexive judgement support psychological stability and enable conversations about physical health to unfold without threatening this stability. The findings suggest that addressing physical health within this relational space requires time and communicative sensitivity, allowing such topics to be taken up in ways attuned to clients’ readiness and fragile motivation.

The data findings also point to the value of dialogic formats that support gradual, client-paced engagement. Approaches such as Motivational Interviewing, with its emphasis on empathy, autonomy support, and eliciting clients’ own reasons for change [47], as well as structured problem-solving methods that help identify small, feasible steps within everyday contexts [48,49], offer concrete conversational tools. These methods can assist in navigating uncertainty and integrating physical-health-related reflections into ongoing relational work, while maintaining alignment with clients’ pace and everyday realities. In relation to the identified logic of restraint, such approaches may help CMH workers move from non-intervention towards gentle activation without undermining psychological stability. By emphasising autonomy, eliciting clients’ own motivations, and working with ambivalence rather than against it, Motivational Interviewing in particular provides a structured way to engage with change while maintaining a non-directive stance. This allows practitioners to remain aligned with the relational and stabilisation-oriented mandate of CMH practice, while still opening space for incremental health-related action.

### 4.6. Secondary Network Level: Everyday Contexts as Sites for Health Experience

At the secondary network level, physical health becomes meaningful when it is embedded in everyday routines and familiar social settings. The findings suggest that the helping relationship often extends into clients’ social environments, reducing overload and translating health-related ideas into everyday relevance. Physical health is taken up more readily when activities are low-threshold, flexible, aligned with clients’ interests, and situated in environments perceived as safe and non-demanding.

Such conditions can be created both within CMH organisations themselves, and in community settings [50], where familiarity and trust provide a starting point for gentle engagement with physical health. From this secure base, clients may extend their participation into broader community offerings, with transitions being most feasible when support or accompaniment is available. Peer-oriented or community-based opportunities for movement, participation, or wellbeing then become experiential spaces in which physical health can be explored at clients’ own pace and without formalised expectations.

### 4.7. Tertiary Network Level: Organisational Structures Enabling Relational Health Work

At the tertiary network level, organisational structures shape how physical health can be taken up in everyday practice. The findings highlight unclear mandates, limited resources, and person-dependent initiatives—patterns that mirror the professional logics of delegation and structural constraint. Extending CMH practice to include physical health requires organisational conditions that recognise it as an adjacent, non-medical component of psychosocial support. Such conditions include clear mandates, time and space for relational health work, reliable interfaces between psychosocial and medical systems, and training opportunities that strengthen CMH workers’ confidence in addressing physical health without altering their professional role. Without this institutional anchoring, health promotion remains dependent on local commitment and cannot become part of routine practice. Without this institutional anchoring, health-related support is likely to remain dependent on local commitment and may not become part of routine practice.

Taken together, the findings indicate that health-promoting practice in CMH is not an additional task but an extension of existing competencies—relational attentiveness, situational judgement, and the ability to contextualise physical health within clients’ everyday networks. When primary, secondary, and tertiary network conditions align, social work in CMH can support clients in shaping physical health within their own contexts, thereby contributing to reductions in avoidable health inequities in a manner consistent with its professional mandate.

From a public health perspective, these findings point to the need for policy frameworks that extend beyond formal mandate clarification to include the provision of stable resources for relational and everyday health work. This may involve funding structures that explicitly recognise time for relationship-based engagement, flexible spaces for low-threshold health-related activities, and organisational support that reduces reliance on individual initiative. Without such structural support, the integration of physical health into CMH practice is likely to remain uneven.

## 5. Conclusions

This study examined how physical health is understood within German CMH practice, which implicit professional logics shape engagement with health-related responsibilities, and what this implies for future work. The findings show that physical health becomes addressable when it can be integrated into relational and stabilising forms of support rather than treated as a separate task.

The five empirically derived logics illustrate how decisions unfold in everyday practice: trusted relationships set the conditions for engagement, psychological stability functions as a primary mandate, responsibilities around physical health are negotiated through recurring acts of boundary-drawing, clients’ fragile motivation requires a stance of restraint, and organisational constraints shape what can be taken up at all. Together, these orientations determine whether, when, and how physical health enters the helping relationship.

The potential for health-promoting practice spans multiple levels of clients’ lives in a networked manner. When helping relationships offer safety, everyday environments provide feasible entry points, and organisational structures create room for relational health work, physical health can be incorporated sustainably into CMH support. From a public health perspective, strengthening these relational, social, and organisational conditions may help reduce avoidable physical health inequalities among people with serious mental illness by enabling everyday support systems to contribute more systematically to health-promoting environments.

## Data Availability

No datasets were generated or analysed during the current study.

## References

[1] WHO (2022). Global Report on Health Equity for Persons with Disabilities.

[2] Braveman P., Arkin E., Orleans T., Proctor D., Acker J., Plough A. (2018). What is Health Equity?. Behav. Sci. Policy.

[3] Marmot M., Bell R. (2019). Social determinants and non-communicable diseases: Time for integrated action. BMJ.

[4] Richter M., Hurrelmann K. (2016). Die soziologische Perspektive auf Gesundheit und Krankheit. Soziologie von Gesundheit und Krankheit.

[5] Dragano N., Gerhardus A., Kurth B.M., Kurth T., Razum O., Stang A., Teichert U., Wieler L., Wildner M., Zeeb H. (2016). Public Health—Mehr Gesundheit für alle. Gesundheitswesen.

[6] Nowak A.C., Kolip P., Razum O. Gesundheitswissenschaften/Public Health. Leitbegriffe Gesundheitsförderung Prävention 2022. https://leitbegriffe.bioeg.de/alphabetisches-verzeichnis/gesundheitswissenschaften-public-health/.

[7] Link B.G., Phelan J. (2014). Stigma power. Soc. Sci. Med..

[8] Druss B.G., Zhao L., Von Esenwein S., Morrato E.H., Marcus S.C. (2011). Understanding Excess Mortality in Persons With Mental Illness: 17-Year Follow Up of a Nationally Representative US Survey. Med. Care.

[9] Walker E.R., McGee R.E., Druss B.G. (2015). Mortality in Mental Disorders and Global Disease Burden Implications: A Systematic Review and Meta-analysis. JAMA Psychiatry.

[10] Chan J.K.N., Tong C.H.Y., Wong C.S.M., Chen E.Y.H., Chang W.C. (2022). Life expectancy and years of potential life lost in bipolar disorder: Systematic review and meta-analysis. Br. J. Psychiatry.

[11] Hjorthøj C., Stürup A.E., McGrath J.J., Nordentoft M. (2017). Years of potential life lost and life expectancy in schizophrenia: A systematic review and meta-analysis. Lancet Psychiatry.

[12] Firth J., Siddiqi N., Koyanagi A., Siskind D., Rosenbaum S., Galletly C., Allan S., Caneo C., Carney R., Carvalho A.F. (2019). The Lancet Psychiatry Commission: A blueprint for protecting physical health in people with mental illness. Lancet Psychiatry.

[13] Nussbaum M.C. (2011). Creating Capabilities: The Human Development Approach.

[14] Sen A. (2001). Development as Freedom.

[15] Sørensen K., Van den Broucke S., Fullam J., Doyle G., Pelikan J., Slonska Z., Brand H. (2012). (HLS-EU) Consortium Health Literacy Project European. Health literacy and public health: A systematic review and integration of definitions and models. BMC Public Health.

[16] Thornicroft G., Deb T., Henderson C. (2016). Community mental health care worldwide: Current status and further developments. World Psychiatry.

[17] Teasdale S.B., Ward P.B., Rosenbaum S., Samaras K., Stubbs B. (2017). Solving a weighty problem: Systematic review and meta-analysis of nutrition interventions in severe mental illness. Br. J. Psychiatry.

[18] Deenik J., Czosnek L., Teasdale S.B., Stubbs B., Firth J., Schuch F.B., Tenback D.E., van Harten P.N., Tak E.C.P.M., Lederman O. (2020). From impact factors to real impact: Translating evidence on lifestyle interventions into routine mental health care. Transl. Behav. Med..

[19] Gühne U., Weinmann S., Riedel-Heller S., Becker T. (2018). Evidenzkapitel: Gesundheitsfördernde Interventionen. S3-Leitlinie Psychosoziale Therapien bei Schweren Psychischen Erkrankungen: S3-Praxisleitlinien in Psychiatrie und Psychotherapie.

[20] IFSW (2014). Global Definition of Social Work—International Federation of Social Workers. https://www.ifsw.org/what-is-social-work/global-definition-of-social-work/.

[21] Gahleitner S.B. (2017). Soziale Arbeit als Beziehungsprofession: Bindung, Beziehung und Einbettung Professionell Ermöglichen.

[22] Ruch G., Turney D., Ward A. (2018). Relationship-Based Social Work: Getting to the Heart of Practice.

[23] Okech V., Neszméry Š., Mačkinová M. (2020). Roles of social workers in mental health care teams: A systematic review of the literature. Proc. CBU Soc. Sci..

[24] Stengler K., Riedel-Heller S.G., Gühne U., Becker T. (2015). Gemeindepsychiatrische Versorgung. PSYCH up2date.

[25] Blumer H., Facsim (1986). Symbolic Interactionism: Perspective and Method.

[26] Kruse J. (2015). Qualitative Interviewforschung: Ein Integrativer Ansatz.

[27] Barbour R. (2018). Doing Focus Groups.

[28] Bohnsack R., Flick U., von Kardorff E., Steinke I. (2003). Gruppendiskussion. Qualitative Forschung.

[29] Pult G. (2024). Präregistrierung: Gesundheitsförderung in der Eingliederungshilfe/Soziale Teilhabe von Menschen mit Schweren Psychischen Erkrankungen: Qualitative Befragung von Klient_innen und Fachkräften. https://osf.io/e7zpf/overview.

[30] Pult G., Frank F. (2026). Barriers and Facilitators to Health Behavior Change: Perspectives of Individuals with Serious Mental Illness in Health-Promoting Interventions—A Systematic Review of Qualitative Evidence. Res. Sq..

[31] Michie S., Ashford S., Sniehotta F.F., Dombrowski S.U., Bishop A., French D.P. (2011). A refined taxonomy of behaviour change techniques to help people change their physical activity and healthy eating behaviours: The CALO-RE taxonomy. Psychol. Health.

[32] Przyborski A. (2004). Gesprächsanalyse und Dokumentarische Methode; Qualitative Auswertung von Gesprächen, Gruppendiskussionen und Anderen Diskursen.

[33] Strauss A., Corbin J. (2010). Grounded Theory: Grundlagen Qualitativer Sozialforschung.

[34] Statistisches Bundesamt Gesundheitspersonal. https://www.destatis.de/DE/Themen/Gesellschaft-Umwelt/Gesundheit/Gesundheitspersonal/_inhalt.html.

[35] Schön D.A. (2017). The Reflective Practitioner.

[36] Schütze F. (1992). Sozialarbeit als „bescheidene“ Profession. Erziehen als Profession: Zur Logik Professionellen Handelns in Pädagogischen Feldern.

[37] Simpson G., Murr A. (2015). The dialectics of change in social work education. J. Pract. Teach. Learn..

[38] Oevermann U. (2001). Zur Analyse der Struktur von sozialen Deutungsmustern. Sozialer Sinn.

[39] Gitterman A., Germain C.B. (2008). The Life Model of Social Work Practice: Advances in Theory and Practice.

[40] Hutchison E.D. (2018). Dimensions of Human Behavior: Person and Environment.

[41] Wendt W.R. (2010). Das Ökosoziale Prinzip: Soziale Arbeit, Ökologisch Verstanden.

[42] Bajraktarov S., Kalpak G., Jovanovic N. (2020). Community mental healthcare: New developments and innovative strategies. Curr. Opin. Psychiatry.

[43] Granovetter M.S. (1973). The Strength of Weak Ties. Am. J. Sociol..

[44] Schmitt M., Holzer B., Stegbauer C. (2019). White, Harrison C. (2008): Identity and Control. How Social Formations Emerge. Princeton: Princeton University Press. Schlüsselwerke der Netzwerkforschung.

[45] Bullinger H., Nowak J. (1998). Soziale Netzwerkarbeit: Eine Einführung für Soziale Berufe.

[46] Thiele G. (2020). Ulrich Otto/Petra Bauer (Hrsg.): Mit Netzwerken Professionell Zusammenarbeiten (Fortschritte der Gemeindepsychologie und Gesundheitsförderung, Bd. 11 und 12) (Bd. I und II als Gesamtwerk im Schuber). Tübingen: Dgvt-Verlag (Deutsche Gesellschaft für Verhaltenstherapie) 2005 (1120 S.) […] [Sammelrezension]. https://www.pedocs.de/frontdoor.php?source_opus=19776.

[47] Miller W.R., Rollnick S. (2012). Motivational Interviewing: Helping People Change.

[48] D’Zurilla T.J., Nezu A.M. (2010). Problem-solving therapy. Handbook of Cognitive-Behavioral Therapies.

[49] Hölzel L.P., Frank F., Bjerregaard F., Areán P.A., Niebling W., Berger M., Bermejo I. (2017). Problemlösetraining in der Primärversorgung—Eine evidenzbasierte Methode für den Versorgungsalltag. Z. Für Allg..

[50] Oettle L. (2025). Breaking Barriers: Identifying and Overcoming Social Exclusion in Voluntary Sports Clubs for Older Adults Affected by Poverty. Phys. Cult. Sport. Stud. Res..

